# Evaluation of Cognitive and Synaptic Restoration in Diabetic Rats Treated With Emblica officinalis, Clitoria ternatea, Vitamin C, and Metformin

**DOI:** 10.7759/cureus.75866

**Published:** 2024-12-17

**Authors:** Ravi Kiran Morampudi, Vishali Neelakandan, Bandarupalli Naveen Kumar, Edward Indla

**Affiliations:** 1 Anatomy, Meenakshi Academy of Higher Education and Research, Chennai, IND; 2 Anatomy, Vels Medical College, Chennai, IND; 3 Anatomy, Mamata Academy of Medical Sciences, Hyderabad, IND; 4 Anatomy, Mamata Medical College, Khammam, IND

**Keywords:** clitoria ternatea, cognitive impairment, diabetes mellitus, emblica officinalis, memory, metformin, morris water maze, spatial learning, synaptic density, vitamin c

## Abstract

Background: Diabetes is known to cause cognitive impairments and synaptic dysfunction. This study investigates the effects of *Emblica officinalis* (EO), *Clitoria ternatea* (CT), Vitamin C, and metformin on cognitive function and synaptic density (SYN) in diabetic rats. This work aims to evaluate the impact of various treatments on spatial learning, memory, and SYN in a diabetic rat model.

Methods: The Morris water maze test was used to assess spatial learning and memory at four time points (Days 1, 3, 14, and 21). SYN was measured using optical density to assess SYN expression. Eight experimental groups were included: Non-diabetic Control, Diabetic Control, Diabetic + EO, Diabetic + CT, Diabetic + Vitamin C, Diabetic + Metformin, Non-diabetic + EO, and Non-diabetic + CT.

Results: On Day 1, the Diabetic Control group exhibited significantly longer latency times, indicating cognitive impairment. Diabetic + EO and Diabetic + CT showed the most significant improvements in cognitive performance compared to other diabetic groups, while Diabetic + Vitamin C and Diabetic + Metformin were less effective. On Day 3, cognitive performance in the diabetic groups improved, but none reached the level of Non-diabetic controls. On Day 14, EO and CT were again the most effective in reducing latency times, followed by Metformin. By Day 21, EO and CT showed significant improvements in cognitive function, with Metformin outperforming Vitamin C. SYN expression was significantly higher in the Non-diabetic + CT and Non-diabetic + EO groups, and these treatments also enhanced SYN expression in diabetic rats, with Metformin showing the greatest improvement.

Conclusion: The results suggest that EO and CT offer significant therapeutic potential for mitigating cognitive deficits and enhancing SYN in diabetic animals. Although metformin also improved cognitive function and SYN, it exhibited the most robust restorative effects. These findings highlight the potential of herbal treatments like EO and CT for managing cognitive dysfunction in diabetes.

## Introduction

Diabetes mellitus is a major metabolic disorder affecting millions worldwide, characterized by elevated blood glucose levels [1], particularly in India, where it affects about 50 million people. It is the third leading cause of death in many developed countries, affecting 6-8% of the general population [2]. Conventional treatments like insulin therapy and oral hypoglycemic drugs can be costly and have side effects [3]. As a result, there is growing interest in medicinal plants as potential therapeutic alternatives for diabetes management. Herbs like *Momordica charantia* L., *Syzygium cumini* (L.) Skeels, and *Ocimum tenuiflorum* L. have shown promise in treating diabetes with minimal side effects [4]. These plant-based remedies offer benefits such as fewer adverse effects, easier availability, and lower costs compared to conventional treatments [1].

Diabetes mellitus, especially type 2 diabetes (T2DM), is closely associated with changes in the structure of the brain and cognitive dysfunction. The pathophysiology of diabetic cognitive impairment is multifactorial, involving hyperglycemia, insulin resistance, and abnormal glucose metabolism in the brain. Insulin resistance, a hallmark of T2DM, is linked to impaired cerebral glucose metabolism and is emphasized as an important pathogenic mechanism. Other contributing factors include oxidative stress, inflammation, mitochondrial dysfunction, and disruptions in various signaling pathways [5]. Cognitive deficits can occur early in diabetes and worsen with disease duration and poor glycemic control. Although the precise mechanisms causing diabetes-related cognitive impairment are complicated, it is believed that aberrant insulin action and poor glucose metabolism play key roles [6].

Recent research has explored natural remedies for managing diabetes-associated cognitive impairments and neurological disorders. Medicinal plants have shown promise due to their antioxidant, anti-inflammatory, and neuroprotective properties. Through a variety of mechanisms, including antioxidant activity, anti-inflammatory actions, and neurotoxicity inhibition, these herbs help alleviate neurological problems brought on by diabetes [7]. Traditional Chinese Medicine has demonstrated effectiveness in treating diabetic cognitive dysfunction by alleviating insulin resistance, microvascular dysfunction, and inflammation [8]. Natural compounds such as curcumin, resveratrol, and berberine are among the most frequently cited in diabetes research [9]. While anti-diabetes methods and neuroprotective natural compounds show promise in reducing brain injury in patients with T2DM, to create focused therapy and preventative measures for diabetes-related cognitive impairment, more study is required [10].

The studies have explored the potential of natural compounds in addressing diabetes-related cognitive decline and neuropathy. *Emblica officinalis* (EO) has shown promising results in attenuating diabetic neuropathic pain by modulating oxidative-nitrosative stress and inflammatory markers in diabetic rats [11]. Likewise, when given early, a mixture of extracts from *Clitoria ternatea* (CT) and *Salacia reticulata* showed protective effects against behavioral and cognitive alterations in young diabetic rats [12]. In diabetic rats, *Murraya koenigii* and EO demonstrated dosage-dependent antioxidant, anti-hyperglycemic, and anti-hyperlipidemic actions; the higher dose (500 mg/kg) showed a notable ability to reverse cognitive impairment [13]. 

CT, a plant used in traditional Indian medicine, has shown promising effects in ameliorating diabetes-induced cognitive decline and neuroprotection in animal studies. Ethanol extracts of CT* *leaves and roots demonstrated antidiabetic and antioxidant properties, improving spatial memory and reducing acetylcholinesterase activity in diabetic rats [14]. The plant extract also exhibited neuroprotective effects on the hippocampal CA3 region and pancreatic tissue in juvenile diabetic rats [15]. Furthermore, CT root extract improved cognitive function and hippocampal long-term potentiation in a rat model of chronic cerebral hypoperfusion, suggesting potential benefits for vascular dementia and Alzheimer's disease [16]. These studies collectively indicate that CT may offer protection against diabetes-induced cognitive decline and warrant further investigation into its mechanisms of action and potential therapeutic applications.

Metformin has shown promising effects in ameliorating cognitive decline associated with diabetes and Alzheimer's disease. In diabetic rat models, metformin treatment improved learning and memory deficits, as assessed by passive avoidance and Y maze tests [17]. Similar cognitive improvements were observed in the SAMP8 mouse model of Alzheimer's disease, with metformin enhancing performance in T-maze footshock avoidance, object recognition, and Barnes maze tasks [18]. In these investigations, spatial learning and memory have been evaluated using behavioral tasks like the Morris water maze (MWM) test [19]. Metformin's beneficial effects on cognition may be attributed to its ability to reduce oxidative stress, improve brain mitochondrial function, and attenuate insulin resistance [20]. At the molecular level, metformin increased protein kinase C levels, decreased phosphorylated tau, and reduced amyloid-β levels in the brain [18]. Despite these encouraging findings, further research is needed to develop effective therapeutic strategies for improving brain function in diabetic individuals and those at risk for Alzheimer's disease [21].

Despite the recognition of cognitive impairments and synaptic loss in diabetes, there is a lack of studies investigating effective treatments that fully restore cognitive function and synaptic density (SYN) to non-diabetic levels. This study aims to address this gap by evaluating the combined therapeutic effects of EO, CT, Vitamin C, and metformin on cognitive function and SYN in diabetic rats, offering insights for potential treatment strategies. By examining the impact of these interventions on both cognitive function and synaptic plasticity, this research offers valuable insights into potential therapeutic avenues for managing cognitive impairments associated with diabetes.

## Materials and methods

Animals

In this investigation, male albino rats weighing 200-250 g were employed. The rats were kept in typical cages with a 12-hour light/dark cycle, a temperature of 20°C, and a humidity of 65%. Rats were fed regular food pellets and water. The institutional ethics committee gave its approval (Ref. No, IAEC-TAEC/VL/07/2022-23) for the treatment and use of all animals in compliance with the guidelines for the treatment and use of laboratory animals. The study was conducted from 16.11.2022 to 15.05.2023.

Preparation of the extracts

The CT flowers and EO fruits (GDC 89) were purchased locally, thoroughly cleaned, and allowed to dry for two weeks in the shade before being ground into a fine powder. Until they were required, the powdered materials were kept in airtight containers. Using a Soxhlet extractor, 500 g of dry, coarsely ground materials were steeped in 1 liter of ethanol for a day before the ethanol evaporated. To enable the residual solvent to evaporate, the extracts employed in this investigation were maintained at 45°C. From the CT and EO, we made alcohol extracts; the yields were 26.48% (w/w) and 29.83% (w/w), respectively.

Drugs

The reference medications utilized in this investigation were vitamin C (Sigma Aldrich Chemicals Pvt Ltd, Hyderabad, India) and diazepam (Calmpose®, 5 mg/tablet, Ranbaxy Laboratories, Gurgaon, India). Tween 80 (0.2%, v/v) was used as the suspending agent when they were administered as suspensions. Analytical-grade solvents were utilized.

Acute oral toxicity study

Each animal was monitored at least twice daily for the course of the 14-day study after receiving plant extracts orally. This was done to assess for changes in grooming, hyperactivity, sedation, corneal reflex, urine, or death. This was done in the course of researching acute oral toxicity.

Diabetes induction

Streptozotocin (STZ) was administered once at a dose of 70 mg/kg of body weight with the aim of causing DM as described earlier [22]. All treated animals were provided with food and drink, and animals that had fasted the night before received intraperitoneal injections. The estimated mean blood glucose levels of the rats were also measured after their blood sugar levels had been allowed to return to normal for four days. Rats with hyperglycemia (blood glucose levels between 250 and 400 mg/dL) and moderate diabetes were used in the study. After STZ medication, blood glucose levels were assessed to make sure the rats developed diabetes before extracts and metformin were given orally. Blood sugar levels were measured at 0, 7, and 21 days following extract and metformin treatments. Every animal received care for 21 days.

Experimental design

**Figure 1:**
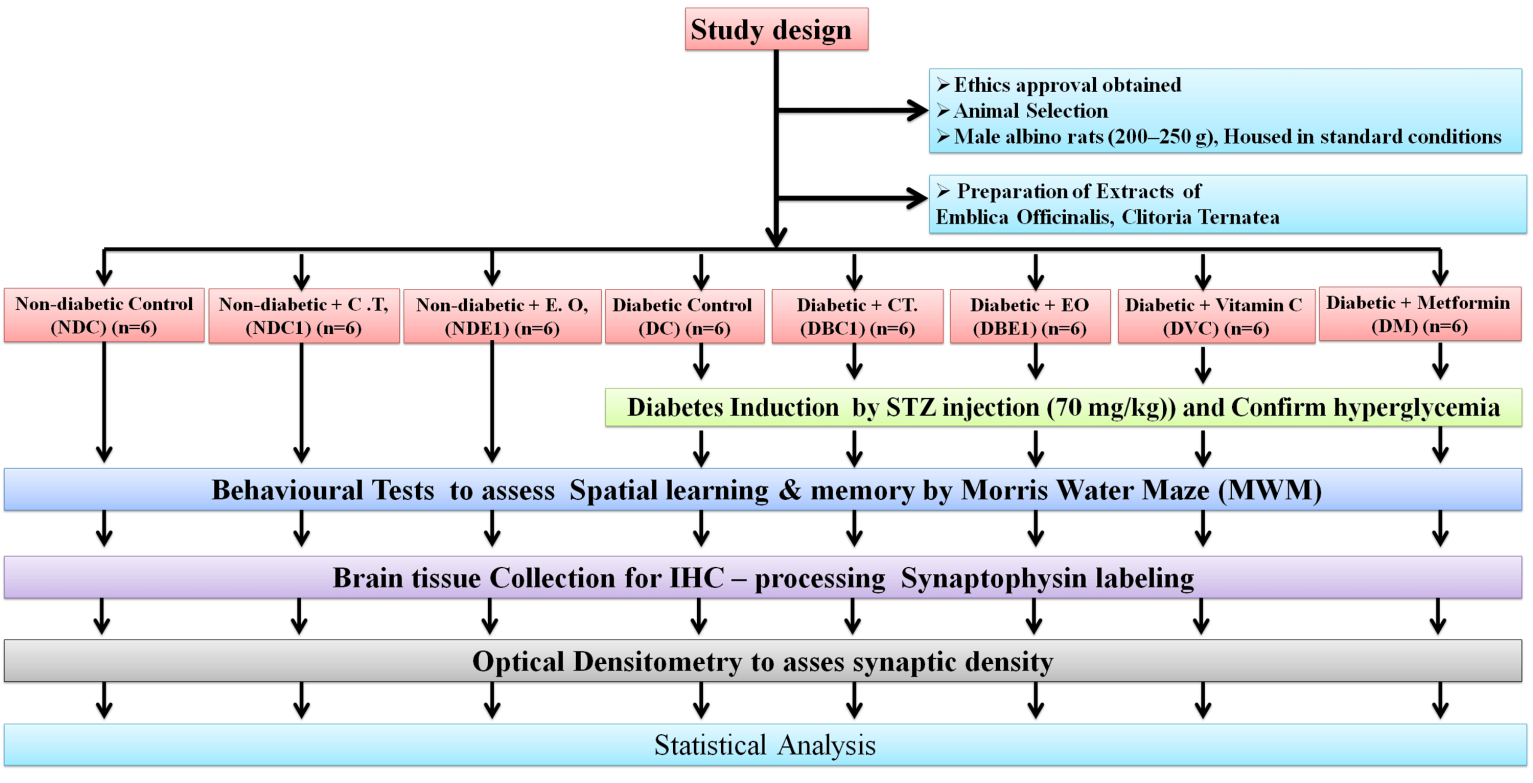
Experimental design. STZ, streptozotocin.

After allowing the animals to acclimate for two weeks, they were randomly assigned to eight equal groups (n = 6): Non-diabetic Control (NDC), Non-diabetic + CT (NDC1), Non-diabetic + EO (NDE1), Diabetic Control (DC), Diabetic + CT (DBC1), Diabetic + EO (DBE1), Diabetic + Vitamin C (DVC), Diabetic + Metformin (DM). This dose was selected based on studies on acute oral toxicity. Each dose of extract was dissolved in 0.5 mL of clean water. The average body weight for each treatment group was recorded at 0, 7, 14, and 21 days. The mean body weight was recorded for each treatment group at 0, 7, 14, and 21 days. To look for any signs of anomalies, the rats were also closely observed during the investigation (Figure 1).

MWM test

**Figure 2:**
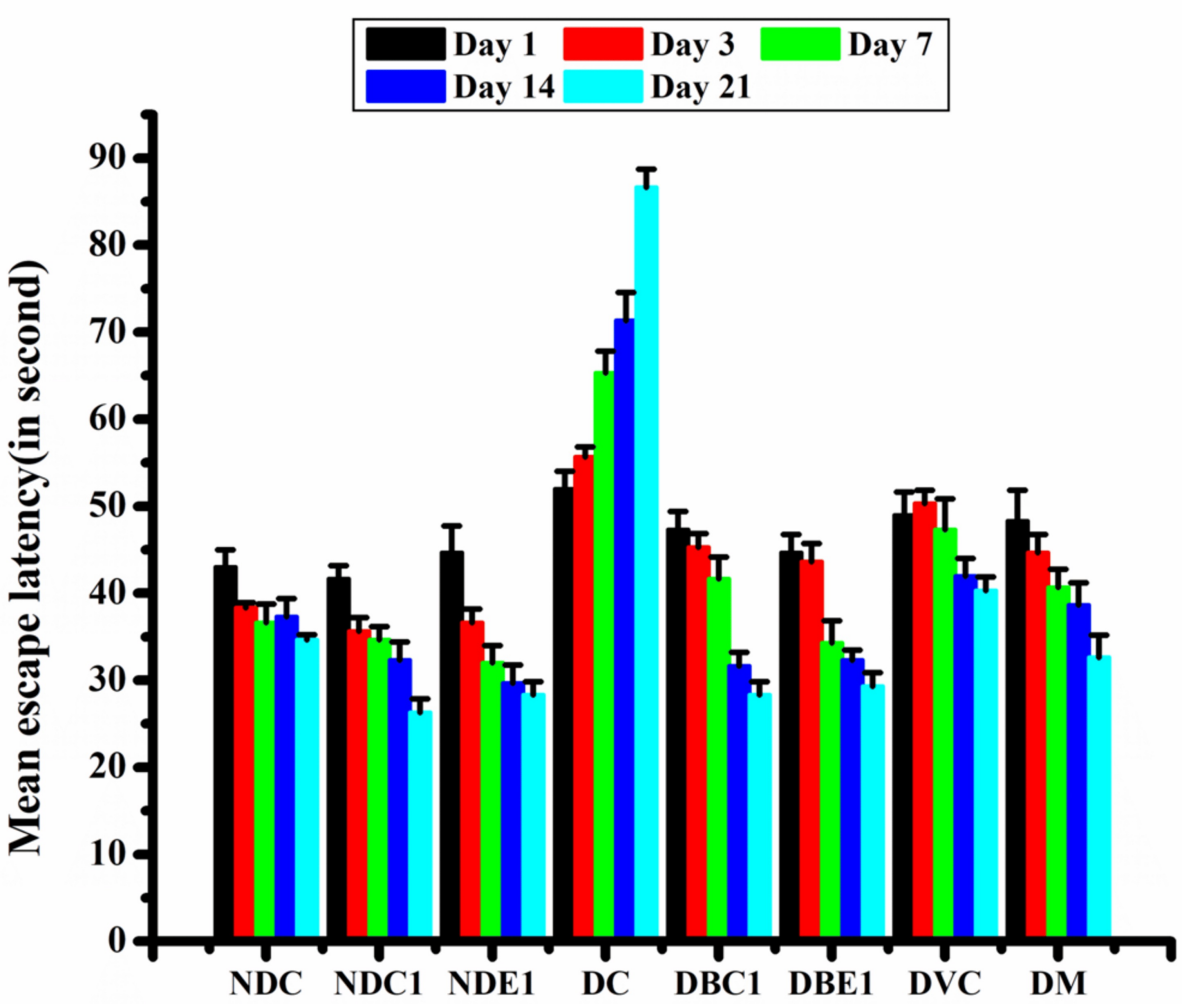
Escape latency of different groups of animals in Morris water maze. The data is expressed as mean ± SD. NDC, Non-diabetic Control; NDC1, Non-diabetic + CT; NDE1, Non-diabetic + EO; DC, Diabetic Control; DBC1, Diabetic + CT; DBE1, Diabetic + EO; DVC, Diabetic + Vitamin C; DM, Diabetic + Metformin; CT, *Clitoria ternatea*; EO, *Emblica officinalis*.

The MWM was used to assess the spatial memory and learning abilities of rats. A 5 cm escape platform was concealed 1 cm below the surface of the 30 cm-deep water in the circular pool of the water maze, which had a 1.5 m diameter and 60 cm height. To make the water opaque, milk was added. The MWM was separated into four equal imaginary quadrants, with the escape platform remaining in the center of the northeast quadrant. The maze had the proper visual cues and was located in a laboratory. For three consecutive days, the rats were trained in daily sessions consisting of three trials separated by a 5-minute intertrial rest. Each trial lasted 120 seconds, during which the rats had to swim to the escape platform. If the rats were unable to locate the platform within 120 seconds, they were physically guided onto it. Every animal was allowed to snooze on the platform for 30 seconds. The site that was furthest distant from the platform was where each experiment started. Transfer/escape latencies were used to quantify the amount of time it took for each rat to find the secret platform. On day 4, the platform was removed from the maze and the rats were allowed to swim in the pool as part of a probing trial. The length of time spent in the target quadrant, which contained the platform in the previous trials, served as a memory test [23,24]. The entire experiment was captured on video, and the tracker video analysis and modeling tool software 5.1.1 (Open Source Physics) was used to monitor the animal movements (Figure 2) as described earlier [23].

Tissue collection and immunohistochemistry evaluation

The PBS (phosphate buffer saline, pH 7.4) was used for transcardial perfusion after rats were sedated with a high dose of halothane. After that, 15% saturated picric acid and 4% PBS-buffered formaldehyde were given. After being dissected, the brains were placed in the fixative and left for 6 hours to postfix. Incubation in citric acid buffer (pH 4.5) for an entire night and then microwave treatment (at 480 W) for 90 seconds were the methods used to retrieve the antigen. After being cryoprotected with PBS containing 30% sucrose and frozen with dry ice, the brain tissues were kept at -80°C until it was examined further. A sliding microtome was used to cut the frozen blocks coronally at a thickness of 30 µm. Six serial slices were obtained from each sample, and they were then cleaned in PBS and stored in an antifreeze solution at -20°C until the next step.

The slices were blocked for an hour at room temperature in PBS with 0.3% triton 10% and normal serum for immunohistochemical labeling after being rinsed three times for 10 minutes each. Sigma-Aldrich's rabbit anti-synaptophysin primary antibody was employed. The slices were incubated at room temperature for the entire night after the primary antibody was diluted in PBS that contained 0.3% Triton X-100 and 2% normal serum. Following three 10-minute PBS washes, every section was incubated for an hour with the biotinylated secondary antibody (diluted 1:500 in PBS containing 0.3% Triton X-100 and 2% NGS). The sections were incubated with HRP (horseradish peroxidase) diluted in PBS for an hour after being rinsed three times for 10 minutes each time. Following three rounds of washing with 0.1 M Tris HCl (pH 7.4), slices were stained for 10 to 15 minutes with 1.25% 3,3-diaminobenzidine and 0.08% hydrogen peroxide. They were then rinsed four times with PBS, mounted, dehydrated, and coverslipped [25,26].

Optical densitometry of synaptophysin immunoreactivity

As previously stated, synaptophysin immunoreactivity was measured using optical densitometry using Image J (NIH, Bethesda, MD) software [25,26]. To put it concisely, digital photomicrographs were captured using a Zeiss Axio microscope and a Carl Zeiss digital camera, the Axiocam MRc5. The exposure times were selected in a way that prevented the pixel brightness from ever being saturated. We measured the pixel brightness of one randomly selected cerebral hemisphere. As a baseline, pixel brightness in the corpus callosum's non-immunoreactive regions was measured. Immunoreactivity to synaptophysin was observed in the hippocampus, as indicated by the averaged background-corrected relative optical densities.

Statistical analysis

The statistical analyses were conducted using SPSS software (version 21; IBM Corp, Armonk, NY). Data are presented as mean ± SEM (standard error of the mean) for normally distributed quantitative variables. For comparing two groups, the Student’s t-test was used, while a one-way analysis of variance (ANOVA) was employed for comparing three or more groups. Tukey’s HSD (honestly significant difference) post hoc test was used for multiple comparisons following ANOVA when homogeneity of variance was assumed. A p-value of <0.05 was considered statistically significant.

## Results

Cognitive function test

**Figure 3:**
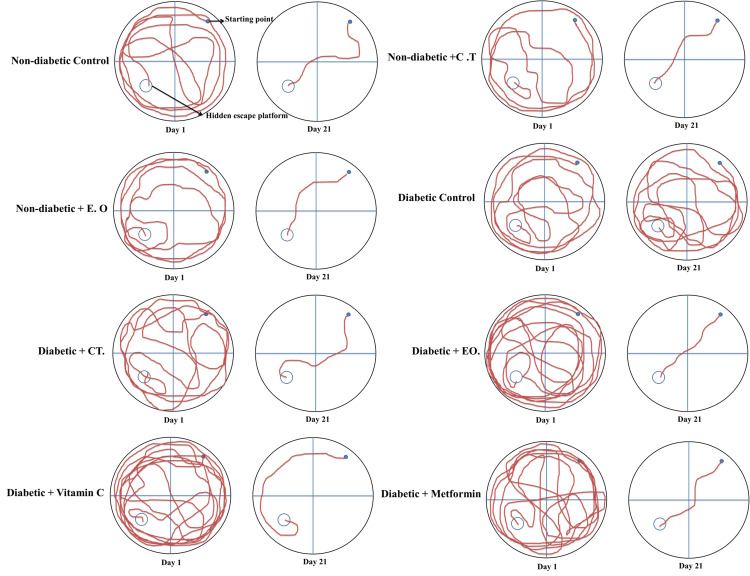
Comparison of animal path tracks of different groups during Morris water maze test. The animal movements were tracked by tracker video analysis and modeling tool software 5.1.1. C.T, *Clitoria ternatea*; E.O, *Emblica officinalis*.

The MWM test was used to evaluate spatial learning and memory across different experimental groups at various time points, revealing the effects of diabetes and various treatments on cognitive function. On Day 1, the Diabetic Control group exhibited the longest latency, indicating significant cognitive impairment compared to the Non-diabetic Control group. Among the diabetic treatment groups, EO showed the most significant improvement in cognitive function, followed by CT, both of which significantly improved memory and learning. Other treatments like Vitamin C and metformin showed some positive effects, but they were less effective than EO and CT in improving cognitive performance (Table 1, Figures 2, 3).

**Table 1:** Comparison of escape latency between different groups CT, *Clitoria ternatea*; EO, *Emblica officinalis*.

Day	Comparison	Group 1 (mean ± SD)	Group 2 (mean ± SD)	t-Statistics	p-Value
Day 1	Non-diabetic Control vs. Diabetic Control	43 ± 2	52 ± 2	9	<0.0001
	Diabetic Control vs. Diabetic + EO	52 ± 2	44.67 ± 2.08	7.185	<0.0001
	Diabetic Control vs. Diabetic + CT	52 ± 2	47.33 ± 2.08	4.572	0.0004
	Diabetic Control vs. Diabetic + Vitamin C	52 ± 2	49 ± 2.65	2.566	0.0224
	Diabetic Control vs. Diabetic + Metformin	52 ± 2	48.33 ± 3.51	3.733	0.0022
	Non-diabetic Control vs. Non-diabetic + CT	43 ± 2	41.67 ± 1.53	1.499	0.1562
Day 3	Non-diabetic Control vs Diabetic Control	38.33 ± 0.58	55.67 ± 1.15	37.975	<0.0001
	Diabetic Control vs. Diabetic + EO	55.67 ± 1.15	43.67 ± 2.08	7.303	<0.0001
	Diabetic Control vs. Diabetic + CT	55.67 ± 1.15	45.33 ± 1.53	4.619	0.0004
	Diabetic Control vs. Diabetic + Metformin	55.67 ± 1.15	44.67 ± 2.08	9.859	<0.0001
	Non-diabetic + CT vs. Non-diabetic + EO	35.67 ± 1.53	36.67 ± 1.53	1.309	0.2115
Day 14	Non-diabetic Control vs. Diabetic Control	37.33 ± 2.08	71.33 ± 3.21	25.111	<0.0001
	Diabetic Control vs. Diabetic + EO	71.33 ± 3.21	32.33 ± 1.15	32.295	<0.0001
	Diabetic Control vs. Diabetic + CT	71.33 ± 3.21	31.67 ± 1.53	31.524	<0.0001
	Diabetic + EO vs. Diabetic + CT	32.33 ± 1.15	31.67 ± 1.53	0.985	0.3415
	Diabetic Control vs. Diabetic + Metformin	71.33 ± 3.21	38.67 ± 2.52	22.632	<0.0001
	Diabetic Control vs. Diabetic + Vitamin C	71.33 ± 3.21	42 ± 2.00	21.915	<0.0001
Day 21	Non-diabetic Control vs. Diabetic Control	34.67 ± 0.58	86.67 ± 2.08	68.084	<0.0001
	Diabetic Control vs. Diabetic + EO	86.67 ± 2.08	32.67 ± 2.52	63.901	<0.0001
	Diabetic Control vs. Diabetic + CT	86.67 ± 2.08	40.33 ± 1.53	63.901	<0.0001
	Non-diabetic + CT vs. Non-diabetic + EO	26.33 ± 1.53	28.33 ± 1.53	2.619	0.0202

On Day 3, the Non-diabetic Control group continued to perform the best, while the Diabetic Control group showed the most severe cognitive deficits. Treatment with EO, CT, and metformin significantly improved the performance in the diabetic groups compared to the Diabetic Control, though none of these treatments fully restored cognitive performance to the level of the non-diabetic group. Notably, EO and CT demonstrated the most promising effects in mitigating cognitive deficits (Table 1, Figure 2).

By Day 14, the Diabetic Control group still exhibited significant cognitive impairment. However, both CT and EO treatments showed the most substantial improvements, significantly reducing latency times compared to the Diabetic Control. While Vitamin C and metformin were also beneficial, they were less effective in improving cognitive performance compared to CT and EO. Despite the improvements, none of the treatments fully restored cognitive function to the levels observed in the Non-diabetic Control group (Table 1, Figure 2).

On Day 21, the Diabetic Control group still showed the greatest cognitive deficits. However, the CT and EO treatment groups continued to show significant improvements in performance. These treatments were more effective than Vitamin C and metformin, with both CT and EO significantly reducing cognitive impairments. Interestingly, CT also appeared to enhance cognitive function in the Non-diabetic Control group, suggesting potential cognitive benefits even in healthy animals (Table 1, Figures 2, 3).

In conclusion, EO and CT were the most effective treatments for improving cognitive function in diabetic animals, although none of the treatments fully restored normal memory and learning abilities. Metformin proved more effective than Vitamin C in mitigating cognitive deficits in diabetes, but CT and EO were the most promising in improving cognitive function overall.

SYN in hippocampus

**Figure 4:**
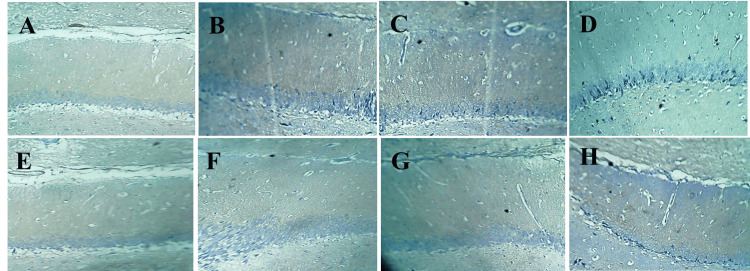
Representative hippocampal sections of different groups stained with anti-synaptophysin antibody. (A) Non-diabetic Control. (B) Non-diabetic + CT. (C) Non-diabetic + EO. (D) Diabetic Control. (E) Diabetic + CT. (F) Diabetic + EO. (G) Diabetic + Vitamin C. (H) Diabetic + Metformin. CT, *Clitoria ternatea*; EO, *Emblica officinalis*.

**Figure 5:**
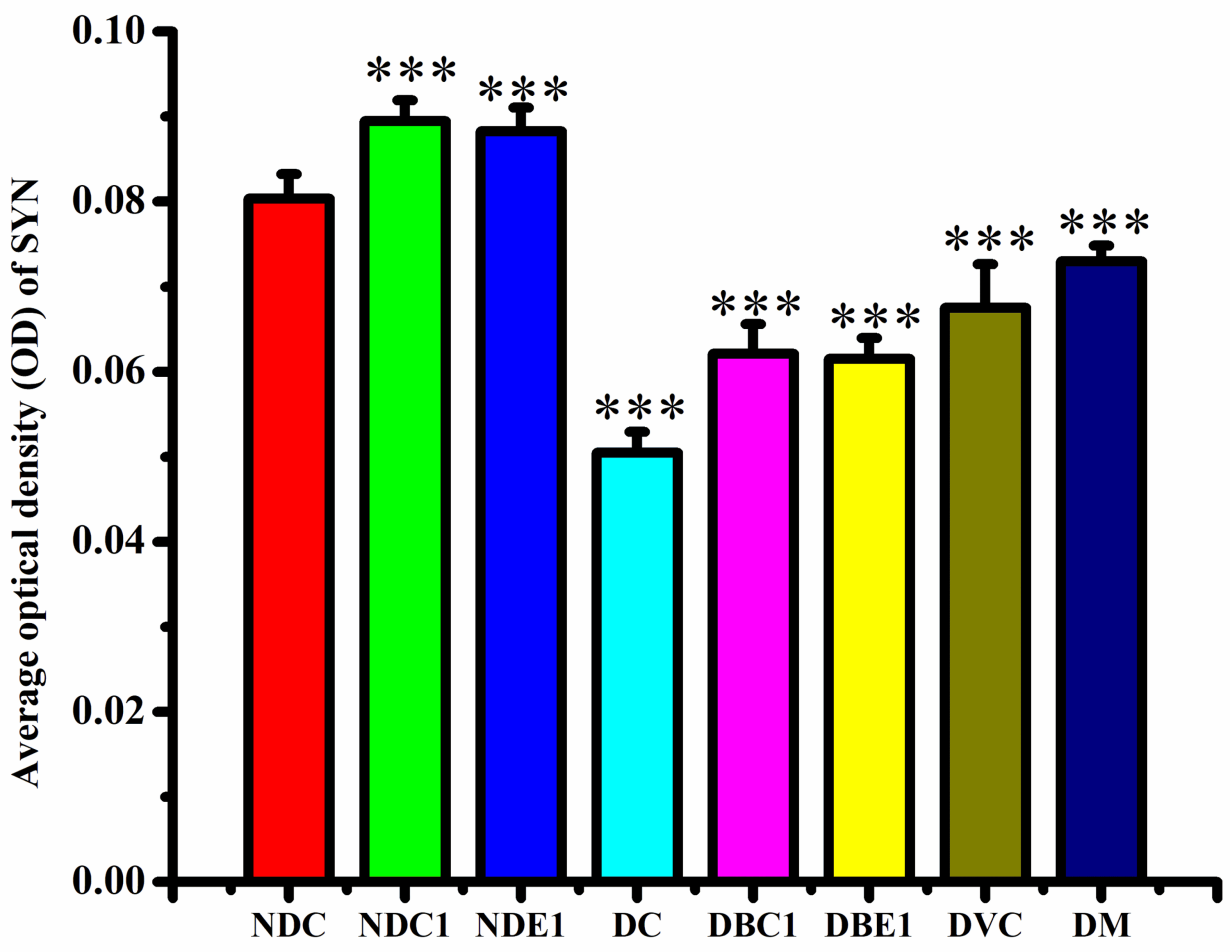
The immunohistochemical expression of average OD of SYN. The data is expressed as mean ± SD. The significance is shown by the superscripted stars (*). OD, optical density; SYN, synaptic density; NDC, Non-diabetic Control; NDC1, Non-diabetic + CT; NDE1, Non-diabetic + EO; DC, Diabetic Control; DBC1, Diabetic + CT; DBE1, Diabetic + EO; DVC, Diabetic + Vitamin C; DM, Diabetic + Metformin; CT, *Clitoria ternatea*; EO, *Emblica officinalis*. ***p=0.001.

The one-way ANOVA results show significant differences in SYN expression across the experimental groups. The Non-diabetic + CT group exhibited the highest SYN expression, followed closely by the Non-diabetic + EO group. In contrast, the Diabetic Control group showed the lowest SYN expression, indicating severe impairment. Treatment with Vitamin C and metformin in the diabetic groups improved SYN expression compared to the Diabetic Control, with metformin showing the most significant restorative effect. The Diabetic + CT and Diabetic + EO groups showed moderate improvements in SYN expression, with metformin demonstrating the strongest therapeutic potential (Table 2, Figures 4, 5).

**Table 2:** Comparison of optical density of SYN between different groups CT, *Clitoria ternatea*; EO, *Emblica officinalis*; SYN, synaptic density.

Comparison	Optical density of SYN (mean ± SD)	Optical density of SYN (mean ± SD)	t-Statistics	p-Value
Non-diabetic + CT vs. Non-diabetic + EO	0.0895 ± 0.0024	0.08825 ± 0.0028	0.947	0.3595
Non-diabetic + CT vs. Diabetic Control	0.0895 ± 0.0024	0.0505 ± 0.0024	31.843	<0.0001
Diabetic + CT vs. Diabetic Control	0.062125 ± 0.0035	0.0505 ± 0.0024	18.187	<0.0001
Diabetic + EO vs. Non-diabetic + CT	0.0615 ± 0.0024	0.0895 ± 0.0024	22.862	<0.0001
Diabetic + Vitamin C vs. Non-diabetic + CT	0.0675 ± 0.0051	0.0895 ± 0.0024	10.951	<0.0001
Diabetic + Metformin vs. Non-diabetic + CT	0.073 ± 0.0019	0.0895 ± 0.0024	15.199	<0.0001
Diabetic + CT vs. Diabetic + EO	0.062125 ± 0.0035	0.0615 ± 0.0024	0.415	0.6843
Diabetic + Vitamin C vs. Diabetic + CT	0.0675 ± 0.0051	0.062125 ± 0.0035	2.453	0.0279
Diabetic + Metformin vs. Diabetic + CT	0.073 ± 0.0019	0.062125 ± 0.0035	7.799	<0.0001
Diabetic + Vitamin C vs. Diabetic + EO	0.0675 ± 0.0051	0.0615 ± 0.0024	2.987	0.0098
Diabetic + Metformin vs. Diabetic + EO	0.073 ± 0.0019	0.0615 ± 0.0024	10.593	<0.0001
Diabetic + Vitamin C vs. Diabetic + Metformin	0.0675 ± 0.0051	0.073 ± 0.0019	2.854	0.0128

Further analysis using t-tests revealed no significant difference in SYN expression between the Non-diabetic + CT and Non-diabetic + EO groups. However, SYN expression was significantly higher in the Non-diabetic + CT group compared to the Diabetic Control group, as well as in the Diabetic + CT group compared to the Diabetic Control. SYN expression in the Diabetic + Metformin group was significantly higher than in the Diabetic Control, though it remained lower than in the Non-diabetic + CT group. Vitamin C also improved SYN expression, but it did not reach the level seen in the Non-diabetic + CT group. Among the diabetic treatments, metformin had the most significant effect on improving SYN expression (Table 2, Figures 4, 5).

Within the diabetic groups, no significant difference in SYN expression was observed between Diabetic + CT and Diabetic + EO. However, Vitamin C and metformin showed significantly higher SYN expression than CT, suggesting they were more effective in enhancing SYN expression in diabetic conditions. Vitamin C was more effective than EO, and metformin had a greater impact on SYN expression than both CT and EO, indicating its stronger therapeutic potential in improving SYN in diabetic conditions (Table 2, Figures 4, 5).

Overall, metformin demonstrated the greatest therapeutic effect in enhancing SYN expression, followed by Vitamin C, while CT and EO also showed positive effects, with CT being the most effective in non-diabetic conditions.

## Discussion

The MWM test revealed significant cognitive impairments in diabetic rats, with the Diabetic Control group showing the longest latency times, indicating severe cognitive deficits. Treatment with EO and CT improved cognitive function, significantly reducing latency times compared to the Diabetic Control group. Vitamin C and Metformin also showed improvements, with metformin being the most effective for synaptic restoration. The Non-diabetic Control group outperformed all other groups. CT and EO were the most promising treatments for mitigating cognitive deficits in diabetes, though none fully restored cognitive function. SYN analysis revealed significant improvements in treated diabetic groups, with metformin showing the most substantial effect, followed by CT and EO, highlighting their therapeutic potential.

The research highlights the complex relationship between diabetes and cognitive impairment, including Alzheimer's disease (AD). Diabetes-induced cognitive dysfunction involves multiple mechanisms, such as abnormal insulin signaling, amyloid-β accumulation, oxidative stress, and inflammation [27]. Brain insulin resistance is increasingly recognized as a pathophysiological factor in AD, with or without metabolic dysfunction [28]. It has been demonstrated that intranasal administering of insulin can improve memory function in people with AD or mild cognitive impairment [28]. The impact of diabetes on cognitive function spans from mild impairment to various forms of dementia, necessitating a standardized diagnostic approach and further research into disease progression and biomarkers [29]. Since insulin resistance and neurodegenerative diseases are increasingly linked, antidiabetic medications may be able to treat dementia [30]. Future research should focus on personalized interventions and longitudinal studies to address the variability in cognitive outcomes among diabetic individuals [29].

Research has shown that diabetes can impair spatial learning, memory, and cognitive function in animal models, particularly in areas like information-processing speed, attention, and cognitive flexibility [31]. Studies using the MWM test have demonstrated that diabetic rats exhibit significant deficits in spatial learning and memory compared to non-diabetic controls This aligns with previous studies that have established the MWM as a reliable measure of spatial learning and memory deficits, particularly in models of diabetes and neurodegenerative diseases [32,33]. Various treatments have been investigated to mitigate these cognitive impairments. Antioxidants such as vitamins C and E, when administered together, have shown promise in improving learning and memory in diabetic rats [34]. Plant-based compounds like crocin from saffron [33] and extracts from *Ficus deltoidea* leaves [32] have also demonstrated potential in ameliorating cognitive deficits and reducing oxidative stress in diabetic animal models. These treatments not only improved cognitive performance but also affected physiological parameters such as blood glucose levels and brain oxidative stress markers [21,32]. These findings suggest that antioxidant and plant-based treatments may offer neuroprotective effects in diabetes-related cognitive impairment.

Both *Murraya koenigii* and EO showed affirm in halting cognitive impairment in diabetic rats by exhibiting dose-dependent antioxidant, anti-hyperglycemic, and anti-hyperlipidemic actions [13], and these findings are in line with the current study findings. However, a systematic review found no strong evidence that any specific diabetes treatment can prevent or delay cognitive impairment in humans with T2DM [35]. The cognitive deficits associated with diabetes, while mild to moderate, may still impact daily activities, especially in demanding situations [31].

In numerous research, CT, also referred to as butterfly pea, has shown notable antidiabetic and antioxidant qualities. The plant's ethanol extract has shown superior antioxidant efficiency, while its methanol extract exhibited a remarkably low IC50 value, indicating strong free radical scavenging ability [36]. In diabetic rats, CT extracts significantly reduced blood glucose levels, with the chloroform extract at 300 mg/kg body weight decreasing glucose from 378.33 mg/dL to 136.33 mg/dL after 12 days of treatment [36]. Recent research has also demonstrated the anxiolytic effects of CT leaf extracts in animal models, with higher doses (300 mg/kg) showing anti-anxiety activity comparable to alprazolam [37], and these findings support the results of the current study.

Research on CT and *Centella asiatica* extracts demonstrates their potential as nootropic agents. Rats treated with CT root extract showed improved passive avoidance learning and memory, correlating with enhanced dendritic arborization in amygdaloid neurons. Similarly, *Centella asiatica* leaf juice administration during rats' growth spurt period resulted in increased dendritic length and branching points in amygdaloid neurons, suggesting memory enhancement properties [38]. *Centella asiatica*, in particular, has shown mitoprotective and antioxidative properties, which may be beneficial in treating neurodegenerative diseases [39]. Furthermore, chronic administration of *Centella asiatica* in rats increased brain-derived neurotrophic factor (BDNF) expression in the prefrontal cortex and improved cognitive performance in the novel object recognition test [40]. These findings support the potential use of these plant extracts in addressing stress-related and neurodegenerative disorders affecting memory and cognition.

Synaptic plasticity characterizes the ability by which synapses can modify the mode of transmission among neurons. SYN is an important protein in presynaptic vesicles. SYN is regarded as an important marker of synaptogenesis and plays an essential role in synaptic plasticity. In the present research, reduced SYN optical density in photomicrographs of SYN expression by immunohistochemical staining was observed in the hippocampus of streptozotocin-induced diabetic rats, which demonstrates a persistent deficit in synaptogenesis and synaptic plasticity. Synaptic plasticity is considered to be an underlying mechanism of cognition [41]. Reduced SYN expression has been shown in animal experiments to be positively associated with memory and learning impairments [42], and AD brains exhibit lower levels of SYN and PSD-95 [43,44].

Recent studies have explored the effects of metformin and other treatments on diabetes and its complications. Metformin, alone or in combination with vitamins C and E, has shown promise in reducing blood glucose levels, improving lipid profiles, and enhancing antioxidant activity in diabetic rats [45]. Metformin has also been found to significantly reduce levels of phospho-Ser129 α-synuclein, a protein associated with Parkinson's disease, through mTOR inhibition and protein phosphatase 2A activation [46]. In comparison with other diabetes medications, metformin remains a primary treatment option, although newer drug classes like SGLT-2 inhibitors have emerged [47]. Additionally, the combination of metformin with micronutrients has shown synergistic effects in alleviating diabetic nephropathy and cardiovascular dysfunction in diabetic rats, suggesting potential benefits as an add-on therapy [48]. These findings highlight the diverse therapeutic potential of metformin in managing diabetes and its associated complications.

Recent studies have demonstrated the antidiabetic potential of CT extract in diabetic rat models. CT extract increased insulin gene expression while decreasing inflammatory markers TNF-α and IL-1β [49]. It exhibited antidiabetic effects through antioxidant and anti-inflammatory mechanisms, improving liver histopathology and enhancing insulin levels [50]. The extract also increased pancreatic CAT (catalase) and SOD (superoxide dismutase) levels while reducing MDA (malondialdehyde), IL-18 (interleukin 18), and glycogen gene expression [50]. Combined extracts of CT leaf and *Trichosanthes dioica* fruit showed superior efficacy in treating diabetes and increasing enzymatic antioxidant activities compared to individual extracts [51]. These findings suggest that CT extract, especially in combination with other plant extracts, holds promise as a potential treatment for diabetes mellitus.

EO has shown promising effects in managing diabetic complications, particularly neuropathy and cataracts. In diabetic rats, EO extracts significantly attenuated behavioral, biochemical, and molecular alterations associated with neuropathic pain by modulating oxidative-nitrosative stress [11]. The plant's broad spectrum of pharmacological activities, including antioxidant, anti-inflammatory, and immunomodulatory properties, is attributed to its polyphenols, especially tannins and flavonoids [52]. These studies consistently demonstrate CT's potential in managing diabetes by modulating antioxidant enzymes, reducing inflammation, and neuroprotective properties, and improving insulin sensitivity. However, none of the studies directly compared CT and EO effects on synaptophysin expression in non-diabetic groups.

While this study provides valuable insights into the effects of treatments on cognitive function and SYN in diabetic conditions, certain limitations highlight areas for future exploration. First, the use of male albino Wistar rats offers a controlled model to standardize results, but expanding to include female subjects or other animal models could provide a more comprehensive understanding of the treatments' effects across different biological systems. The use of a well-established methodology, such as the MWM and immunohistochemistry, provides reliable data but also opens avenues to integrate advanced imaging or molecular techniques for deeper mechanistic insights. These limitations underline opportunities for broader application and refinement in future research.

## Conclusions

The results from the MWM test and SYN measurements highlight significant cognitive deficits and synaptic impairments in diabetic animals compared to non-diabetic controls. The Diabetic Control group exhibited the most severe cognitive and synaptic impairments across all time points in the MWM test, with longer latency times and lower synaptic expression. Among the treatments, EO and CT showed the greatest potential for improving cognitive and synaptic function, with both significantly reducing latency times and enhancing synaptic expression in diabetic animals. Although metformin and Vitamin C also improved both cognitive and synaptic function, metformin demonstrated the most robust restorative effects, outperforming other treatments in both aspects.

Overall, while none of the treatments fully restored cognitive function or SYN to non-diabetic levels, the findings suggest that EO and CT are promising in mitigating cognitive deficits in diabetes, with metformin being the most effective for synaptic restoration. These results emphasize the therapeutic potential of these treatments for managing cognitive impairments associated with diabetes.
